# Blood pressure determinants of target organ damage among adults with white coat hypertension with a focus on nocturnal hypertension and blood pressure dipping

**DOI:** 10.1038/s41371-026-01152-7

**Published:** 2026-04-24

**Authors:** Ionas Papasotiriou, Sotiria Spiliopoulou, Gerasimos Barlas, Damianos Dragonas, Konstantinos Rizogiannis, Efstathios Manios

**Affiliations:** 1 Department of Clinical Therapeutics, Faculty of Medicine, National and Kapodistrian University of Athens, Alexandra Hospital, Athens, Greece; 2 Department of Electrical and Computer Engineering, American University in Dubai, Dubai, United Arab Emirates

**Keywords:** Hypertension, Vascular diseases

## Abstract

White coat hypertension (WCH) is still a grey zone regarding its management by clinicians, while its link to target organ damage (TOD) is still understudied. The aim of this study was to explore the impact of non-dipping status and nocturnal hypertension on TOD in untreated WCH subjects. A cross-sectional study of 547 individuals with WCH was conducted at a single institution. Patients underwent a thorough clinical examination, including medical history, office BP measurements, echocardiography, carotid artery ultrasonography, serum creatinine level measurements, and ambulatory blood pressure monitoring. Left ventricular hypertrophy (LVH), intima-media thickness of the common carotid artery (IMT-CCA) and estimated Glomerular Filtration Rate (eGFR) were used as TOD indices. The patients were divided into dippers and non-dippers, as well as nocturnal hypertensives and normotensives. Among WCH patients, 49.9% were non-dippers and 33.6% had nocturnal hypertension. Dippers had a significantly lower risk of left ventricular hypertrophy (OR: 0.68, 95%CI: 0.48 – 0.97) and higher mean expected eGFR values (b = 7.03, 95%CI: 3.12 – 10.94) than non-dippers. For IMT-CCA, office systolic and diastolic BP were the only significant predictors (b = 0.02, 95%CI: 0.00 – 0.03 and b = -0.03, 95%CI: -0.05 – -0.01). Office systolic BP was also significantly associated with eGFR (b = -1.68, 95%CI: -2.84 – -0.52). The results of this study indicate that non-dipping status is a significant risk factor for LVH and renal function in WCH subjects. Therefore, nighttime BP patterns, especially non-dipping, should be examined in patients with WCH.

## Introduction

White coat hypertension (WCH) is a well-established clinical entity characterized by persistently elevated blood pressure (BP) in a physician’s office but normal BP in out-of-office settings [1, 2]. Much of the existing literature suggests that WCH is a benign condition. However, several studies have indicated that individuals with WCH present an intermediate risk of target organ damage (TOD) and cardiovascular morbidity between that of normotensive and hypertensive subjects [3]. Therefore, a meticulous and multiangle approach should be considered when treating these patients.

Moreover, in all blood pressure (BP) phenotypes, BP normally follows a circadian rhythm, which is characterized by a 10% to 20% decrease during sleep compared to daytime. Subjects who do not demonstrate this diurnal pattern (less than 10% BP fall) are classified as non-dippers [4, 5]. Hypertensive patients that belong to the aforementioned group are at higher risk of developing hypertension-mediated target organ damage (TOD) and cardiovascular disease (CVD) than dippers [5]. Moreover, apart from the dipping status, it has been reported that isolated nocturnal hypertension per se, is a distinct indicator for increased overall mortality compared with normotension [6]. Consequently, both fluctuations and absolute values of nocturnal BP are significant factors for the incidence of CVD and TOD in patients with hypertension [4, 7].

However, the potentially detrimental effects of non-dipping or nocturnal hypertension on TOD have not been fully elucidated in patients with WCH. Therefore, this study aimed to investigate the impact of non-dipping status and nocturnal hypertension on left ventricular hypertrophy (LVH), intima-media thickness of the common carotid artery (IMT-CCA), and renal function in adults with WCH.

## Materials and methods

### Participants

A consecutive series of 2607 adults who were referred for evaluation at the Hypertension Center of our Department between January 2015 and January 2024, and underwent office BP measurements, 24-h ambulatory BP monitoring, echocardiographic and carotid ultrasonographic measurements, and blood biomarker assessment, were initially screened. The study population fulfilled the following inclusion criteria: 1) no history or clinical evidence of hypertension-related complications (coronary artery disease, heart failure, cerebrovascular disease, or peripheral artery disease); 2) no clinical signs or laboratory evidence of secondary causes of arterial hypertension; and 3) no previous antihypertensive treatment. Risk factors such as diabetes mellitus, hyperlipidemia, smoking history, and body mass index were recorded.

A total of 547 patients demonstrated elevated office (SBP ≥ 140 mmHg or/and DBP ≥ 90 mmHg) and normal 24-h ambulatory BP values (24-h SBP < 130 mmHg and 24-h DBP < 80 mmHg) and were defined as WCHs. The study population was divided into dippers (SBP nocturnal fall >10%) and non-dippers (SBP nocturnal fall <10%) as well as into nocturnal hypertensives (nighttime SBP ≥ 120 mmHg or/and nighttime DBP ≥ 70 mmHg) and nocturnal normotensives (nighttime SBP < 120 mmHg and nighttime DBP < 70 mmHg).

These individuals were referred to our laboratory by their primary physicians for evaluation of previous office BP alterations or symptoms suggesting possible abnormal BP variations, such as headache, tinnitus, dizziness, fainting, and epistaxis. The study was approved by the local scientific committee, and all participants provided informed consent.

### Office and ambulatory BP measurements

Office BP was measured in both arms using a clinically validated automated sphygmomanometer (Omron 705IT; Omron Healthcare, Kyoto, Japan) by a physician. An appropriate cuff was applied around the non-dominant arm, and three BP measurements were taken at 1-min intervals. The last two recordings were averaged to obtain a single systolic and diastolic office BP value. During the measurements, the participants remained seated with their arms supported and placed at the level of the heart.

 All participants underwent 24-h ambulatory BP monitoring on a usual working day. They were instructed to act and work as usual and to keep their non-dominant arm still and relaxed at their side during the measurements. Ambulatory BP was recorded using oscillometric Spacelabs 90207 equipment (SpaceLabs, Redmond, WA). Readings were obtained automatically at 20-min intervals throughout the 24-h study period. Daytime was defined as the interval between 09:00 h and 21:00 h and nighttime as the interval between 01:00 h and 06:00 h [8]. For each patient, the mean 24-h, daytime, and nighttime SBP and DBP were calculated. The degree of SBP dipping was calculated as (1 – daytime SBP/nighttime SBP) * 100. All participants were instructed to rest and sleep at night and maintain their usual activities during the day. None of the study participants were bedridden or hospitalized during the ambulatory BP monitoring.

### Echocardiographic measurements

A complete M-mode two-dimensional and color-flow Doppler echocardiographic examination was performed for each patient. Studies were performed using a commercially available Hewlett Packard machine (Sonos 1000; Hewlett Packard, Andover, Massachusetts, USA) and a 2.5 MHz phased-array transducer. Echocardiographic pulsed Doppler data were recorded at a sweep speed of 50 or 100 mm/s using a Panasonic AG-7350 videocassette recorder (model AG-MD830E; Panasonic, Osaka, Japan) during four to six cardiac cycles and stored on high-quality super VHS videotape for later playback and blinded analysis by the same two investigators experienced in echocardiography [9, 10]. M-mode echocardiography measurements of the left ventricular posterior wall, septum, and left ventricular internal diastolic and systolic dimensions at the level of the chordae tendineae were obtained. The mean value of all measurements of the left ventricle per observer was computed. LVM was calculated using the Devereux formula according to the Penn Convention Protocol [11], and the LVM index (LVMI) was calculated as LVM divided by the body surface area (LVM/BSA). The presence of left ventricular hypertrophy (LVH) was determined if LVMI > 115 g/m^2^ for men or >95 g/m^2^ for women [12].

### Carotid ultrasonography measurements

The left and right common carotid arteries (CCA) were examined using a high-resolution ultrasound Doppler system (Acuson 128XP) with a 7 MHz linear array transducer in three directions: anterolateral, posterolateral, and mediolateral. CCT imaging was performed by a single experienced sonographer who was blinded to the patients’ profiles. The intima-media thickness (IMT) was calculated as the mean of the right and left IMT of the CCA. Mean values were calculated from ten measurements on each side, taken 10 mm proximal to the carotid bifurcation, following the M-line method [13].

### Renal function

Renal function was evaluated using the estimated Glomerular Filtration Rate (eGFR) and the 2021 CKD-EPI creatinine equation [14], as proposed by the National Kidney Foundation and the American Society of Nephrology [15]. eGFR was calculated for 371 participants with available serum creatinine levels.

### Statistical analysis

Descriptive statistics were used to present a basic description of the study participants, where quantitative variables are presented as mean (standard deviation) and qualitative variables as absolute frequencies (%). Participants were categorized into dipper/non-dipper and with/without nocturnal hypertension groups. To compare the baseline characteristics between groups, an independent samples t-test was used for quantitative variables and the chi-square test for qualitative variables. To explore the association between demographic, clinician, and BP variables, both simple and multiple logistic or linear regression analyses were performed, where a hierarchical modeling approach was used for multivariate analysis. For LVH, a hierarchical logistic regression model was performed, where age, sex, smoking status, BMI, diabetes mellitus, and hyperlipidemia were inserted into the model with the Enter method as established confounders. Then, BP parameters (office SBP, office DBP, mean 24 h SBP, mean 24 h DBP, mean nighttime SBP, mean nighttime DBP, nocturnal hypertension and dipping status) were evaluated as possible predictors using the stepwise selection process. For IMT-CCA and eGFR, the same hierarchical approach was followed using the linear regression models. All statistical analyses were performed using STATA 18.0 for Windows, and statistical significance was set at a level of α = 0.05.

## Results

The percentages of non-dippers and nocturnal hypertensives in the WCH subjects were 49.9% and 33.6%, respectively. Detailed demographic, clinical, and BP parameters are presented in Table 1. Dippers and non-dippers, as well as nocturnal hypertensives and normotensives, did not differ significantly in terms of baseline demographic and clinical characteristics. In this sample of WCH adults, non-dippers had significantly lower office SBP, office DBP, 24 h SBP and dipping percentage than dippers (p < 0.05, Table 1). In contrast, WCH adults with nocturnal hypertension presented significantly higher 24 h SBP, 24 h DBP, nighttime SBP, nighttime DBP, and dipping percentage compared to those with nocturnal normotension (p < 0.001, Table 1).

**Table 1:** Demographic characteristics and blood pressure parameters according to dipping status and nocturnal hypertension in white-coat hypertensive subjects.

	WCH (n = 547)					
	Dippers (n = 274)	Non-Dippers (n = 273)	p-value	Nocturnal NT (n = 363)	Nocturnal HTN (n = 184)	p-value
***Baseline characteristics***						
Age, years, mean (SD)	53.8 (12.3)	54.5 (13.2)	0.511	54.8 (12.9)	53.0 (12.3)	0.115
Male gender, No (%)	105 (38.3)	106 (38.8)	0.903	132 (36.4)	79 (42.9)	0.136
Body mass index, kg/m^2^, mean (SD)	27.7 (4.3)	27.7 (4.2)	0.894	27.6 (4.3)	27.9 (4.2)	0.459
Type 2 Diabetes, No (%)	16 (5.8)	25 (9.2)	0.141	25 (6.9)	16 (8.7)	0.448
Hyperlipidemia, No (%)	102 (37.2)	103 (37.7)	0.903	144 (39.7)	61 (33.2)	0.137
Smoking, No (%)	84 (30.7)	82 (30.0)	0.875	105 (28.9)	61 (33.2)	0.310
***Blood pressure parameters, mean (SD)***						
Office SBP, mmHg, mean (SD)	148.7 (14.7)	146.1 (15.2)	**0.040**	147.3 (14.9)	147.8 (15.1)	0.661
Office DBP, mmHg, mean (SD)	91.8 (9.5)	90.1 (9.5)	**0.035**	90.7 (9.7)	91.5 (9.0)	0.336
24-h SBP, mmHg, mean (SD)	118.6 (7.1)	120.2 (6.5)	**0.007**	117.7 (7.0)	122.9 (5.0)	**<0.001**
24-h DBP, mmHg, mean (SD)	72.2 (5.4)	72.4 (5.2)	0.724	70.7 (5.1)	75.6 (4.0)	**<0.001**
Daytime SBP, mmHg, mean (SD)	125.5 (7.6)	122.4 (6.8)	**<0.001**	123.2 (8.0)	125.4 (5.7)	**0.001**
Daytime DBP, mmHg, mean (SD)	77.6 (5.8)	74.8 (5.6)	**<0.001**	75.3 (6.1)	77.9 (4.8)	**<0.001**
Nighttime SBP, mmHg, mean (SD)	107.1 (7.1)	116.3 (6.9)	**<0.001**	108.3 (7.3)	118.4 (6.1)	**<0.001**
Nighttime DBP, mmHg, mean (SD)	63.3 (5.8)	68.3 (5.4)	**<0.001**	62.9 (4.9)	71.4 (4.0)	**<0.001**
Systolic Dipping, No (%)	14.6 (3.6)	5.0 (3.6)	**<0.001**	12.0 (5.4)	5.5 (4.8)	**<0.001**

*WCH* white-coat hypertension, *SBP* systolic blood pressure, *DBP* diastolic blood pressure, *LVMI* left ventricular mass index.

Results from simple logistic regression showed that a higher BMI was associated with significantly higher odds of LVH (OR: 1.05, 95%CI: 1.01 – 1.09, Table 2, model 1), while dipping was associated with 31% lower odds of LVH than non-dipping status (OR: 0.69, 95%CI: 0.49 – 0.97, Table 2, model 1). The multiple logistic regression model showed that older age (OR: 1.02 per 10-year increase, 95%CI: 1.00 – 1.03) and BMI (OR: 1.07, 95%: 1.02 – 1.11) were associated with significantly higher odds of LVH (Table 2, model 2), while among BP parameters, only dipping status was a significant factor for LVH, where dippers had 32% (OR: 0.68, 95%CI: 0.48 – 0.97) lower odds of LVH than non-dippers (Table 2, model 2).

**Table 2:** Simple and multiple logistic regression analyses of factors associated with left ventricular hypertrophy in white-coat hypertensive adults.

	Left Ventricular Hypertrophy (n = 547)			
	Model 1		Model 2	
***Covariates***	***OR (95%CI)***	***p-value***	***OR (95%CI)***	***p-value***
Age	1.01 (0.99 – 1.03	0.074	1.02 (1.00 – 1.03)	0.045
Male sex	0.49 (0.35 – 0.70)	**<0.001**	0.44 (0.30 – 0.64)	**<0.001**
Current smoking	0.94 (0.65 – 1.36)	0.756	1.21 (0.82 – 1.80)	0.340
BMI	1.05 (1.01 – 1.09)	**0.026**	1.07 (1.02 – 1.11)	**0.004**
Type 2 Diabetes	1.23 (0.64 – 2.38)	0.539	1.18 (0.60 – 2.32)	0.640
Hyperlipidemia	0.89 (0.63 – 1.26)	0.509	0.78 (0.54 – 1.14)	0.782
***BP parameters***	***b (95%CI)***	***p-value***	***b (95%CI)***	***p-value***
Office SBP	1.00 (0.99 – 1.02)	0.543	-	-
Office DBP	0.99 (0.97 – 1.01)	0.230	-	-
24-h SBP	1.02 (0.99 – 1.04)	0.154	-	-
24-h DBP	0.98 (0.95 – 1.02)	0.300	-	-
Daytime SBP	1.01 (0.99 – 103)	0.413	-	-
Daytime DBP	0.98 (0.95 – 1.01)	0.113	-	-
Nighttime SBP	1.02 (0.99 – 1.04)	0.067	-	-
Nighttime DBP	1.00 (0.98 – 1.03)	0.865	-	-
Nocturnal hypertension	1.35 (0.93 – 1.94)	0.111	-	-
Dipping	0.69 (0.49 – 0.97)	**0.033**	0.68 (0.48 – 0.97)	**0.032**

*OR* odds ratio, *CI* confidence interval, *BMI* body mass index, *BP* blood pressure, *SBP* systolic blood pressure, *DBP* diastolic blood pressure.

Simple linear regression analysis showed that among covariates, age, male gender, and hyperlipidemia were significantly associated with IMT-CCA (Table 3, model 1), while among BP parameters office systolic and diastolic BP, 24 h systolic and diastolic BP, daytime systolic and diastolic BP and nighttime SBP were significantly associated with IMT-CCA (Table 3, model 1). Results from multiple linear regression showed that age (b = 0.06 per 10-year increase, 95%CI: 0.05 – 0.07), male sex (b = 0.6, 95%CI: 0.03 – 0.09) and BMI (b = 0.01, 95%CI: 0.00 – 0.02) were significantly and positively associated with IMT-CCA. Among the BP parameters, office SBP (b = 0.02, 95%CI: 0.01 – 0.03) and DBP (b = -0.03, 95%CI: -0.05 – -0.01) were significantly associated with IMT-CCA.

**Table 3:** Simple and multiple linear regression analyses of factors associated with left intima media thickness of the common carotid artery in white-coat hypertensive adults.

	Intima-Media Thickness of the Common Carotid Artery (n = 547)			
	Model 1		Model 2	
***Covariates***	***b (95%CI)***	***p-value***	***b (95%CI)***	***p-value***
Age^*^	0.06 (0.05 – 0.07)	**<0.001**	0.06 (0.05 – 0.07)	**<0.001**
Male sex	0.05 (0.02 – 0.08)	**0.002**	0.06 (0.03 – 0.09)	**<0.001**
Current smoking	0.00 (-0.03 – 0.03)	0.957	0.02 (-0.01 – 0.05)	0.182
BMI	0.01 (-0.00 – 0.02)	0.197	0.01 (0.00 – 0.02)	**0.040**
Type 2 Diabetes	0.03 (-0.03 – 0.09)	0.267	0.01 (-0.05 – 0.05)	0.990
Hyperlipidemia	0.03 (0.01 – 0.07)	**0.032**	0.01 (-0.03 – 0.04)	0.934
***BP parameters***	***b (95%CI)***	***p-value***	***b (95%CI)***	***p-value***
Office SBP^**^	0.02 (0.00 – 0.03)	**<0.001**	0.02 (0.01 – 0.03)	**<0.001**
Office DBP^**^	-0.03 (-0.05 – -0.01)	**<0.001**	-0.03 (-0.05 – -0.01)	**<0.001**
24-h SBP^**^	0.05 (0.02 – 0.07)	**<0.001**	-	-
24-h DBP^**^	-0.04 (-0.06 – -0.01)	**0.017**	-	-
Daytime SBP^**^	0.04 (0.02 – 0.06)	**0.001**	-	-
Daytime DBP^**^	-0.04 (-0.07 – -0.01)	**0.003**	-	-
Nighttime SBP^**^	0.03 (0.01 – 0.05)	**0.001**	-	-
Nighttime DBP^**^	-0.02 (-0.04 – 0.01)	0.204	-	-
Nocturnal hypertension	-0.01 (-0.05 – 0.02)	0.393	-	-
Dipping	-0.01 (-0.04 – 0.02)	0.407	-	-

b: regression coefficient, CI: confidence interval, BMI: body mass index, BP: blood pressure, SBP: systolic blood pressure, DBP: diastolic blood pressure.

^*^by each 10-year increase.

^**^by each 10-mmHg increase.

For eGFR, simple regression analysis showed that age, hyperlipidemia, and office SBP were significantly associated with eGFR (Table 4, model 1). The multivariate model showed that among covariates, only age had a significant and negative association with eGFR (b = -6.71 per 10-year increase, 95%CI: -8.07 – -5.34), while among BP parameters, office SBP had a negative association (b = -1.68, 95%: -2.84 – 0.52) with eGFR, and dippers had a significantly higher mean expected eGFR (b = 7.03, 95%CI: 3.12 – 10.94) than non-dippers (Table 4, model 2).

**Table 4:** Simple and multiple linear regression analyses of factors associated with estimated glomerular filtration rate in white-coat hypertensive adults.

	Estimated Glomerular Filtration Rate (n = 371)			
	Model 1		Model 2	
***Covariates***	***b (95%CI)***	***p-value***	***b (95%CI)***	***p-value***
Age^*^	-7.10 (-8.43 – -5.77)	**<0.001**	-6.71 (-8.07 – -5.34)	**<0.001**
Male sex	-0.32 (-4.16 – 3.52)	0.869	-2.57 (-5.99 – 0.66)	0.139
Current smoking	3.84 (-0.16 – 7.83)	0.060	1.03 (-2.55 – 4.60)	0.573
BMI	0.18 (-0.27 – 0.63)	0.443	0.14 (-0.26 – 0.53)	0.505
Type 2 Diabetes	-6.85 (-13.96 – 0.27)	0.059	-4.05 (-10.24 – 2.14)	0.199
Hyperlipidemia	-6.01 (-9.74 – -2.29)	**0.002**	-2.67 (-6.00 – 0.66)	0.116
***BP parameters***	***b (95%CI)***	***p-value***	***b (95%CI)***	***p-value***
Office SBP^**^	-1.74 (-2.98 – -0.49)	**0.006**	-1.68 (-2.84 – -0.52)	**0.005**
Office DBP^**^	0.75 (-1.21 – 2.70)	0.454	-	-
24-h SBP^**^	0.13 (-2.53 – 2.79)	0.923	-	-
24-h DBP^**^	3.75 (0.19 – 7.31)	**0.039**	-	-
Daytime SBP^**^	0.05 (-2.39 – 2.50)	0.966	-	-
Daytime DBP^**^	0.34 (0.02 – 0.66)	**0.036**	-	-
Nighttime SBP^**^	0.19 (-1.99 – 2.37)	0.867	-	-
Nighttime DBP^**^	2.07 (-0.95 – 5.10)	0.178	-	-
Nocturnal hypertension	0.54 (-3.49 – 4.56)	0.794	-	-
Dipping	3.53 (-0.17 – 7.23)	0.062	7.03 (3.12 – 10.94)	**<0.001**

b: regression coefficient, *CI* confidence interval, *BMI* body mass index, *BP* blood pressure, *SBP* systolic blood pressure, *DBP* diastolic blood pressure.

^*^by each 10-year increase.

^**^by each 10-mmHg increase.

## Discussion

This study aimed to examine an understudied domain in hypertension research, specifically, to explore the impact of nighttime BP patterns, namely non-dipping and nocturnal hypertension, on TOD in patients with WCH. Our results showed that non-dipping status arose as a significant predictor of cardiac and renal organ damage, but not of subclinical carotid atherosclerosis and that it seems to be a stronger indicator of left ventricular hypertrophy than nocturnal hypertension in untreated WCH subjects. These data indicate that BP non-dipping is a more significant risk factor associated with TOD than nocturnal hypertension.

O’Brien et al. (1988) were the first to introduce the terms “dipper” and “non-dipper” [16], and since then, several studies have demonstrated the adverse effects of non-dipping on TOD in hypertensive patients [17–20]. However, the superiority of the non-dipping status over other BP parameters is not always consistent. Cuspidi et al. [2012], after studying 343 patients, concluded that in the presence of nocturnal hypertension, the difference in dipping status was not a strong modifying factor of LVMI [6]. These findings suggest that nocturnal hypertension is a better indicator of TOD than non-dipping in hypertensive patients. Nonetheless, a previous study conducted by Perez-Lloret et al. (2008) in 223 patients also supports the above conclusions [21]. Another observational study of 682 subjects in Korea confirmed the “domination” of nocturnal hypertension as a better predictor than non-dipping for TOD –left ventricular hypertrophy in specific–, especially in relatively high CVD risk patients [22]. Similar results have also been shown among a pediatric population (5-18 years old) with primary hypertension [23].

However, the interpretation of our findings in patients with WCH is unclear, since the available published data is insufficient for drawing certain conclusions. Turfaner et al. studied the effect of dipping status on LVMI in WCH patients [24]. Although LVMI was slightly increased in non-dippers, the difference was not statistically significant. Presumably, the small number of subjects (90 patients in total) was not sufficient to exhibit significant differences between the two groups [24]. In contrast, true hypertension, both nocturnal hypertension and non-dipping, has been associated with a worse CVD risk profile and exacerbated TOD [25, 26]. A wide variety of prospective observational studies have concluded that nocturnal BP is a better predictor of worse prognosis than daytime or 24-h ambulatory BP [26–28]. Non-dipping is also associated with higher levels of organ damage, poor cardiovascular prognosis, and mortality [29–31].

In summary, as far as left ventricular hypertrophy and renal function are concerned, our results support that non-dipping is a better predictor than nocturnal hypertension in WCH, while according to existing literature, in hypertensive patients, this observation is not always consistent. This could be attributed to the fact that nocturnal hypertension is not common in patients with ambulatory BP values within the normal range. In contrast, non-dipping is commonplace even in patients with normal daytime and nocturnal BP values, since it is defined as the ratio between daytime and nighttime BP. Another explanation, although vague, could be that different pathogenetic pathways are implicated in each condition, and as a result, differentiated clinical outcomes are observed.

The clinical implication of our study lays on the current management of WCH. TOD and CVD play a crucial role in the clinical approach and treatment regimens for these patients, since many studies have indicated that WCH is of clinical importance [32, 33]. However, our results imply that the identification of a non-dipper markedly increases the risk of TOD; therefore, these patients should be evaluated for TOD and observed more strictly. Given the increased significance of nocturnal BP patterns as a predictor of outcome, the clinical management of WCH should consider not only the mean 24-h ambulatory BP values but also daytime and nighttime BP values. However, given the limitations of our study, more evidence is needed to better shape current clinical practice. These include the cross-sectional design, which does not allow examining the prospective effect of BP parameters on cardiovascular outcomes, but also the lack of other organ damage, like the brain and peripheral arteries. Nevertheless, the use of ABPM for assessing BP phenotypes has been criticized by some experts [34]. In addition, the use of fixed time intervals to define daytime and nighttime BP, although in accordance with the suggestions of the European Society of Hypertension, may present another limitation of the study [8].

## Conclusions

This study showed that in patients with WCH, non-dipping leads to a higher risk of LVH and reduced renal function, indicating that dipping status should be considered in the management of this BP phenotype. We also suggest that future studies should investigate more thoroughly the differentiation of the effect of nocturnal hypertension and dipping status on organ damage at both clinical and molecular levels. Moreover, the management of these patients should be elucidated. Future perspectives of this study should focus on randomized controlled trials so that evidence-based conclusions can be drawn.

## Summary

### What is known about topic

- White coat hypertension remains a grey zone regarding clinical management
- Limited evidence exists about the role of nocturnal blood pressure patterns on organ damage in this type of hypertension

### What this study adds

- Here we examined the predictive value of a wide variety of blood pressure parameters on target organ damage
- Dipping status emerged as the most significant predictor of cardiovascular damage in white coat hypertension

## Data Availability

The data used in this study are not publicly available. However, the STATA code used for statistical analysis is available upon reasonable request.
